# Interleukin-27 Ameliorates Atherosclerosis in ApoE^−/−^ Mice through Regulatory T Cell Augmentation and Dendritic Cell Tolerance

**DOI:** 10.1155/2022/2054879

**Published:** 2022-11-11

**Authors:** Wenbin Xu, Ruirui Zhu, Zhengfeng Zhu, Kunwu Yu, Yue Wang, Yan Ding, Jian Yu, Hongxia Tang, Qiutang Zeng, Yucheng Zhong

**Affiliations:** ^1^Department of Cardiology, Union Hospital, Tongji Medical College, Huazhong University of Science and Technology, China; ^2^Hubei Key Laboratory of Biological Targeted Therapy, Union Hospital, Tongji Medical College, Huazhong University of Science and Technology, China; ^3^Hubei Provincial Engineering Research Center of Immunological Diagnosis and Therapy for Cardiovascular Diseases, Union Hospital, Tongji Medical College, Huazhong University of Science and Technology, China; ^4^Department of Infectious and Immunological Diseases, Medical & Health Center for Women and Children, Tongji Medical College, Huazhong University of Science and Technology, China; ^5^Department of Cardiovascular Surgery, Union Hospital, Tongji Medical College, Huazhong University of Science and Technology, China

## Abstract

Atherosclerosis, which is characterized by chronic inflammation in the arterial wall, is driven by immune cells and cytokines. Recent evidence indicated that interleukin (IL)-27 showed pleiotropic properties in immune diseases. However, precise mechanisms of IL-27, especially in atherosclerosis remains unknown. In our research, we examined the influence of the administration of IL-27 and an anti-IL-27p28 antibody (anti-IL-27p28-Ab) on both the initiation and the progression of atherosclerosis. In the groups (both the initiation and the progression) receiving recombinant IL-27 administration, the formation of atherosclerotic plaques was suspended, and the percentage of regulatory T cells (LAP^+^ or Foxp3^+^) in the spleen and peripheral blood was increased. Meanwhile, the number of T helper 1 (Th1) and T helper 17 (Th17) cells was decreased. In the peripheral blood plasma, TGF-*β* and IL-10 expression were increased, while the levels of IFN-*γ* and IL-17 were reduced. As for lesions, the mRNA expression of Foxp3, TGF-*β*, and IL-10 was increased, while that of IFN-*γ* and IL-17 was reduced. In the anti-IL-27p28 antibody groups, we obtained opposite results. We also observed that DCs treated with IL-27 display a tolerogenic phenotype and that IL-27–treated tolerogenic DCs (tDCs) are likely to play a protective role during atherosclerosis. Our study indicates that IL-27 or adoptive transfer of IL-27 loaded tDCs may be a new therapeutic approach in atherosclerosis.

## 1. Introduction

IL-27 is a member of the IL-12 family and is composed of Epstein-Barr virus-induced gene3 (EBI3) and p28 subunits, which are produced by active antigen-presenting cells [1]. IL-27 binds to IL-27ra and gp-130 complex, which is expressed on multiple cell types, including T lymphocytes [2]. IL-27 has diverse immunoregulatory activities under physiological and pathological conditions. It has been reported that IL-27 performs an anti-inflammatory function by acting on conventional CD4^+^ T cells to induce IL-10-producing cells that are implicated in controlling inflammatory responses [3]. Meanwhile IL-27 exerts proinflammatory effects by promoting Th1, CD8, natural killer (NK), T follicular helper (Tfh), and B cell proliferation/functions and by inhibiting Treg and Th2 cell generation in parasites infection [4]. However, study on the effects of IL-27/anti-IL-27 antibody (anti-IL-27p28-Ab) administration to atherosclerosis model in ApoE^−/−^ mice has not been reported.

Atherosclerosis is a lipid-driven chronic inflammatory disease characterized by progressive atherosclerotic plaque growth. Modified low-density lipoproteins are accumulated in the arterial wall. Infiltrating monocytes take up the modified low-density lipoproteins and become foam cells, which make plaques unstable, resulting in acute myocardial infarction [5]. Many studies have indicated that numerous immune cells promote chronic inflammation and progressive plaque growth [4]. Further studies have shown that Th1 cells promote atherosclerosis initiation and progression [6, 7]. However, the role of Th2 cells in atherosclerosis remains controversial. Importantly, recent studies have shown that the importance of type 17 T helper cells (Th17 cells) and regulatory T cells (Tregs) in the pathogenesis of various immune disorders, particularly in atherosclerosis.

It is well known that Tregs play a significant role in maintaining peripheral tolerance, preventing autoimmune diseases, and restraining chronic inflammatory diseases. Tregs produce cytokines, including TGF-*β* [8] and IL-10 [9], which can suppress the development and characteristics of atherosclerotic plaques. Treg deletion accelerates the development of atherosclerosis, and adoptive transfer Treg suppresses atherosclerosis [10]. Classic CD4^+^ Tregs are identified by the intracellular marker Foxp3 [11]. Targeting classic Tregs is limited by the detection of Foxp3 and surface Treg markers on activated cells. Other types of Tregs, including Tr1 and Th3 cells, have also been described, though they are not well characterized as classic Foxp3^+^ Tregs. LAP, which is the aminoterminal domain of the TGF-*β* precursor peptide, remains noncovalently associated with the TGF-*β* peptide after cleavage and forms the latent TGF-*β* complex. It has been reported that the latency-associated peptide (LAP)-positive Treg subset has greater in vivo suppressive properties than the classic Foxp3^+^ Treg subset in an experimental autoimmune encephalomyelitis model and functions in a TGF-*β*-dependent manner [12]. The effector T cell response is suppressed by CD4^+^LAP^+^Tregs in a TGF-*β*-dependent fashion both ex vivo and in vivo. We recently uncovered that the percentage of naturally occurring CD4^+^CD25^+^Tregs and CD4^+^LAP^+^Tregs are reduced in patients with acute coronary syndrome (ACS) [13] and that the number and function of CD4^+^LAP^+^Tregs are decreased in patients with dilated cardiomyopathy [14]. More importantly, we reported that LAP^+^ Tregs induced by nasal oxidized low-density lipoprotein [15] or thymic stromal lymphopoietin [16] attenuated atherosclerosis in apolipoprotein E-deficient (ApoE^−/−^) mice. All these results suggest that LAP^+^ Tregs can also be regulated by external factors. Exploring these regulatory factors is particularly important.

Dendritic cell is one of the professional antigen-presenting cells, which administrate immune response and peripheral tolerance [17–19]. It has been reported that secretion of inflammatory cytokines was suppressed by IL-10-treated DCs, and these DCs induced generation of regulatory T cells (Tregs) [20, 21]. More and more evidence showed that specific antigen-loaded tDCs alleviate atherosclerosis and experimental autoimmune myocarditis [22, 23]. Of note, our previous results indicated that IL-37 plus Troponin I-loaded DCs acquired the characteristics of tolerogenic DCs (tDCs) and increased the number of Tregs [24]. Surprisingly, in murine, adoptive transfer of these tDCs significantly ameliorated inflammatory cells infiltration in the infarct heart [24]. However, the utility of immunoregulatory tDCs as therapy for atherosclerosis is just starting.

Given these findings, we investigated whether the administration of IL-27 or an anti-IL-27p28 antibody alleviates atherosclerosis. We found that the administration of IL-27 suppressed the initiation and progression of atherosclerotic plaques in ApoE^−/−^ mice. IL-27 promoted the generation of LAP^+^ Tregs and Foxp3^+^ Tregs which suppress effector T cell responses. We have further observed that IL-27-tDCs induce a tolerogenic immune response and alleviate atherosclerosis in ApoE^−/−^ mice.

## 2. Methods

### 2.1. Animals

Male C57BL/6 mice and ApoE^−/−^ mice on a C57BL/6 background (The Jackson Laboratory) were bred and maintained in the Animal Center of Beijing University. The mice were kept in a specific pathogen-free facility (Tongji Medical College) and were fed a normal chow diet or a Western-type diet containing 0.15% cholesterol and 21% fat. The mice were 6 weeks old at the start of the experiment divided into six groups (*n* = 6 mice/group). Among 166, 500, and 1500 ng/mL these three concentrations, 500 ng/mL IL-27 was the optimal one. The treatment of 500 ng/mL IL-27 resulting in significantly decreased atherosclerotic lesion size, robust upregulation of LAP^+^ and Foxp3^+^ Treg frequencies, and inhibition of Th1 and Th17 frequencies. Therefore, we used 500 ng/mL IL-27 in this study. As for anti-IL-27p28 antibody, we also investigated three concentrations (16.6, 50, and 150 *μ*g/mL) and 50 *μ*g/mL anti-IL-27p28 antibody was the optimal one (Figure S1). Groups A, B, and C received intraperitoneal injections of recombinant IL-27 (BioLegend, San Diego, CA; 500 ng every three days), an anti-IL-27p28 antibody (anti-IL-27p28-Ab; eBioscience, San Diego, CA; 50 *μ*g every three days), or phosphate-buffered saline (PBS) (0.1 mL every three days) for 8 weeks, while groups D, E, and F started treatment after 8 weeks of Western-type diet consumption and were treated for 8 weeks. Stumhofer and Hunter reported that gp-130 and IL-27ra complex, which binds to IL-27p28, plays an essential role in IL-27 signaling, and therefore, we used anti-IL27p28-Ab as an antagonist of IL-27 signaling [3]. Diet and water were administered ad libitum. All experiments were carried out in compliance with the guidelines of the Care and Use of Laboratory Animals (Science and Technology Department of Hubei Province, China).

### 2.2. Cell Culture and DC/T-Cell Coculture

Bone marrow–derived DCs (BMDCs) were generated with GM-CSF and IL-4 as previously described [24]. On day 3, nonadherent cells were washed and refed the same concentrations of GM-CSF and IL-4. On day 6, half of the culture medium was replaced with fresh medium. Immature DCs (imDCs) were obtained after 8 days of culture. CD4^+^ T cells were isolated from splenocytes of male C57BL/6 mice by a CD4 MicroBead mouse kit: Miltenyi Biotec. After that, imDCs, tDCs, or mDCs (2 × 10^5^ cells/mL) and CD4^+^ T cells (1 × 10^6^ cells/mL) were cocultured. The mixed cells were cultured for 3 days at 37°C with 5.0% CO_2_ in 2 mL RPMI 1640 supplemented with 10% FCS.

### 2.3. Weight and Lipids Measurements

The weight of each mouse was recorded at 2, 4, 6, and 8 weeks following the start of the intraperitoneal injection. Blood was collected into heparin tubes. The plasma was isolated from mouse blood by centrifugation at 1,200 g for 10 minutes. Total cholesterol, high-density lipoprotein cholesterol, and triglyceride plasma levels were measured by enzymatic assay and determined with an autoanalyzer (Hitachi 917).

### 2.4. Atherosclerotic Lesion and Heart Tissue Analysis

Atherosclerotic lesions in the aortic sinus and descending thoracic aorta were quantified. In brief, the heart including the aortic roots, which are parallel to the atria, was prepared, and sections were fixed in 4% formaldehyde, processed, and embedded in optimum cutting temperature (OCT) compound. Five- to seven-micrometer sections of the aortic sinus were cut at 35 *μ*m intervals starting from the 3-valve cusps. In addition, the descending thoracic aorta was dissected, fixed, opened longitudinally, and pinned onto black wax plates. All the above specimens were stained with Oil Red O and hematoxylin. For plaque area measurement in each mouse, Image-Pro Plus 6.0 (Media Cybernetics) was used.

### 2.5. Real-Time PCR

Total RNA was extracted with TRIzol reagent (Invitrogen, Carlsbad, CA) according to the manufacturer's instructions and converted into cDNA using the PrimeScript RT Reagent Kit (TaKaRa, Shiga, Japan). A 10 *μ*l PCR mixture contained 1 *μ*g of total cDNA and 5 pmol each of the primers that were purchased from Applied Biosystems (Foster City, CA). All reactions were performed with SYBR Premix Ex TaqII (TaKaRa) and incubated in an Applied Biosystems 7500 Real-Time PCR system (Bio-Rad, Singapore) in a 96-well plate according to the manufacturer's protocol. The primer sequences used in this study are listed in Table 1. For each sample, the mRNA expression level was normalized to the GAPDH mRNA level.

## 3. Flow Cytometric Analysis of Th1 Cells, Th17 Cells, Foxp3^+^ Tregs, and LAP^+^ Tregs

### 3.1. Analysis of Foxp3^+^ Tregs and LAP^+^ Tregs

The mononuclear cells from the spleen were isolated with Ficoll-Paque PLUS (MP Biomedicals), and the erythrocytes were removed. PBMCs and the mononuclear cells from the spleen were washed with PBS and then resuspended at a density of 2 × 10^6^ cells/mL in completed culture medium (RPMI-1640 medium supplemented with 10% heat-inactivated fetal calf serum; Gibco BRL, Grand Island, NY). The cell suspension was seeded in 24-well culture plates. For LAP induction, the cells were stimulated with soluble anti-CD3 and anti-CD28 antibodies (2 *μ*g/mL each; eBioscience) for 24 h. The incubator was set to 37°C and a 5% CO_2_ environment. For Treg analysis, the PBMCs and the mononuclear cells from the spleen were stained with anti-mouse CD4- FITC-conjugated (eBioscience), anti-mouse CD25- APC-conjugated (eBioscience), and anti-mouse LAP- PerCP-Cy5.5-conjugated (BioLegend) antibodies for 30 min at 4°C. Anti-mouse IgG1-PE-conjugated (eBioscience) isotype controls were used to enable normalization and confirm antibody specificity. After surface staining, the cells were washed with PBS, fixed and permeabilized with a fixation/permeabilization reagent (eBioscience) at 4°C for 30 min, washed twice in a 1 × permeabilization buffer (eBioscience), and incubated with an anti-mouse Foxp3-PE-conjugated (eBioscience) at 4°C for 30 min.

### 3.2. Analysis of Th1 and Th17 Cells

For Th cell analysis, PBMCs and the mononuclear cells from the spleen were seeded at a density of 2 × 10^6^ cells/well in RPMI-1640 medium supplemented with 10% heat-inactivated fetal calf serum (Gibco BRL) and stimulated with PMA (50 ng/mL; Sigma, St. Louis, MO) plus ionomycin (1 *μ*g/mL, Sigma), and monensin (500 ng/mL, eBioscience) for 4 h at 37°C under 5% CO_2_ conditions. After stimulation, the cells were washed with PBS, incubated with an anti-mouse CD4- FITC-conjugated antibody (eBioscience) at 4°C for 30 min, washed with PBS, fixed with an IC fixation buffer (eBioscience) at 4°C for 30 min, washed twice in a 1 × permeabilization buffer and stained with anti-mouse IFN-*γ* PE-conjugated (eBioscience), or anti-mouse IL-17A-PE-conjugated (eBioscience) antibodies at 4°C for 30 min. Anti-mouse IgG1- PE-conjugated and anti-rat IgG2- PECy7-conjugated (eBioscience) isotype controls were used to enable normalization and confirm antibody specificity.

Flow cytometric acquisition was performed using a FACSCalibur (BD Immunocytometry Systems, San Jose, CA), and all analyses were performed using FlowJo software (FlowJo, LLC, Ashland, OR).

### 3.3. Cytokine Detection

Plasma levels of TGF-*β*, IFN-*γ,* and IL-17 were measured by enzyme-linked immunosorbent assay (ELISA) according to the manufacturer's instructions (NeoBioscience Co., China). The intraassay and interassay variation coefficients for all ELISAs were <10%. All samples were measured in duplicate.

### 3.4. Statistical Analysis

Values are expressed as the mean ± SD in the text and figures. Student's *t*-test was used to detect differences between two groups and one-way ANOVA followed by Newman-Keuls post hoc test was used for comparisons ≥3 groups. A probability value of *P* < 0 · 05 was considered statistically significant.

## 4. Results

### 4.1. The Initiation of Atherosclerosis Was Inhibited by Recombinant IL-27 and Promoted by Anti-IL-27p28 Antibody

**Figure 1:**
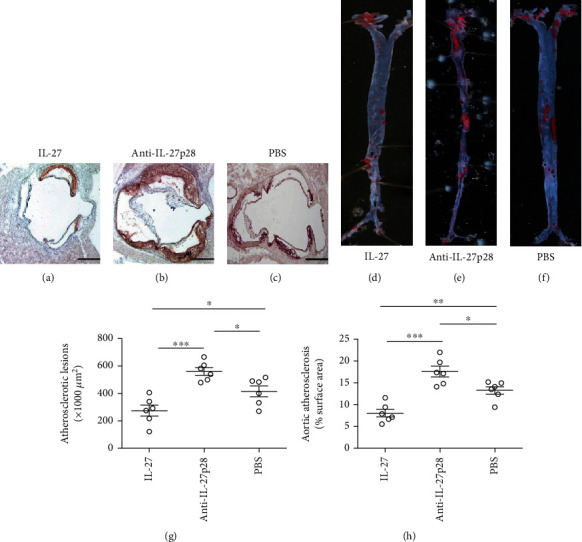
Recombinant IL-27 inhibited the initiation of atherosclerosis. (a), (b), and (c), Representative photomicrographs show Oil Red O and hematoxylin stained aortic root sections from groups treated with IL-27, anti-IL-27p28-Ab, or PBS while they were fed a high-fat diet for 8 weeks, respectively. (g) Data from group (a), (b), and (c) are shown. A black bar represents 200 *μ*m. (d), (e), and (f) Representative photomicrographs show Oil Red O stained the descending aortas from groups treated with IL-27, anti-IL-27p28-Ab, or PBS while they were fed a high-fat diet for 8 weeks, respectively. (h) Data from group (d), (e), and (f) are shown. ^∗∗∗^*P* < 0.001, ^∗∗^*P* < 0.01, ^∗^*P* < 0.05.

To determine whether the administration of recombinant IL-27 affects the initiation of atherosclerosis, we treated 6-week-old ApoE^−/−^ mice with IL-27/anti-IL-27p28-Ab/PBS while they were fed a high-fat diet for 8 weeks. Compared with the mice treated with PBS, the mice treated with IL-27 in the early stage of atherosclerosis exhibited a 33.7% reduction in the aortic sinus atherosclerotic lesion size (mean aortic sinus plaque area 415.3 ± 40 × 10^3^ *μ*m^2^ vs 275.2 ± 40 × 10^3^ *μ*m^2^, respectively; *P* = 0.03; Figure 1). The mice treated with the anti-IL-27p28 antibody in the early stage of atherosclerosis exhibited a 35.4% increase in aortic sinus atherosclerotic lesion size than those treated with PBS (mean aortic sinus plaque area 415.3 ± 40 × 10^3^ *μ*m^2^ vs 562.2 ± 28 × 10^3^ *μ*m^2^, respectively; *P* = 0.013; Figure 1). Furthermore, we measured aortic plaque burden via en face analysis. Similar results are shown in representative images of Oil Red O staining. Compared to the PBS-treated mice, the mice treated with IL-27 in the early stage of atherosclerosis showed a significant 39.2% reduction in aortic plaque burden (Figure 1; 13.28 ± 0.88% vs 8.08 ± 0.87%, respectively; *P* = 0.002), while those treated with anti-IL-27p28-Ab showed a 32.2% increase in aortic plaque burden (Figure 1; 13.28 ± 0.88% vs 17.55 ± 1.20%, respectively; *P* = 0.017).

### 4.2. The Progression of Atherosclerosis Was Inhibited by Recombinant IL-27 and Promoted by Anti-IL-27p28 Antibody

**Figure 2:**
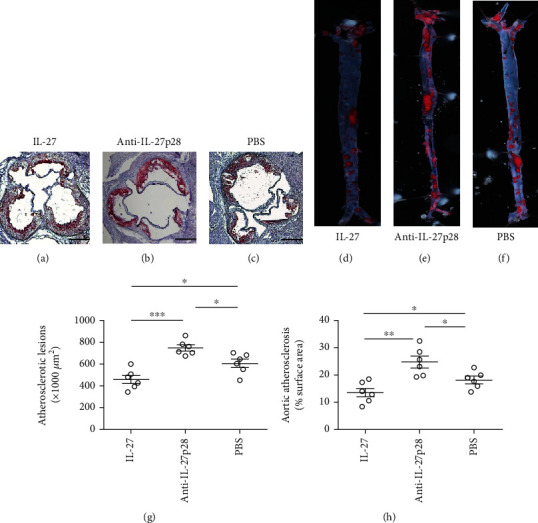
Recombinant IL-27 inhibited the progression of atherosclerosis. (a), (b), and (c), Representative photomicrographs show Oil Red O and hematoxylin stained aortic root sections from groups treated with IL-27, anti-IL-27p28-Ab, or PBS for another 8 weeks after 8 weeks of western diet consumption, respectively. (g) Data from group (a), (b), and (c) are shown. A black bar represents 200 *μ*m. (d, e, f), representative photomicrographs show Oil Red O stained the descending aortas from groups treated with IL-27, anti-IL-27p28-Ab, or PBS for another 8 weeks after 8 weeks of western diet consumption, respectively. (h) Data from group (d), (e), and (f) are shown. ^∗∗∗^*P* < 0.001, ^∗∗^*P* < 0.01, ^∗^*P* < 0.05.

To determine whether the administration of recombinant IL-27 affects the progression of atherosclerosis, we treated ApoE^−/−^ mice with IL-27/anti-IL-27p28 antibody/PBS for another 8 weeks after 8 weeks of western diet consumption. Using groups modeling the progression of atherosclerosis, we obtained results similar to those observed with the early stage groups. Compared with the mice treated with PBS, the mice treated with IL-27 during the progression of atherosclerosis displayed a reduction of 24.4% in aortic sinus atherosclerotic lesion size (605.7 ± 38 × 10^3^ *μ*m^2^ vs 457.8 ± 37 × 10^3^ *μ*m^2^, respectively; *P* = 0.02; Figure 2). The mice treated with the anti-IL-27p28 antibody were substantially increased by 23.6% in aortic sinus atherosclerotic lesion size (605.7 ± 38 × 10^3^ *μ*m^2^ vs 748.7 ± 27 × 10^3^ *μ*m^2^, respectively; *P* = 0.012; Figure 2) than those treated with PBS. We also measured aortic plaque burden via en face analysis in these groups. Compared with the mice treated with PBS, the mice treated with IL-27 during the progression of atherosclerosis exhibited a reduction of 24.4% in aortic sinus atherosclerotic lesion size (18.03 ± 1.3% vs 13.45 ± 1.6%, respectively; *P* = 0.047; Figure 2), while the mice treated with the anti-IL-27p28 antibody showed an increase of 23.6% in aortic sinus atherosclerotic lesion size (18.03 ± 1.3% vs 24.73 ± 2.1%, respectively; *P* = 0.021; Figure 2).

### 4.3. Effect of Recombinant IL-27 on Body Weight and Plasma Lipid Levels

No adverse effect was observed to be frequently associated with the treatments, and body weight and plasma lipid profiles were unaffected. These findings indicate that the administration of recombinant IL-27 or the anti-IL-27p28 antibody does not markedly affect body weight and plasma total cholesterol levels.

### 4.4. The Generation of Foxp3^+^ and LAP^+^ Tregs in ApoE^−/−^ Mice Was Elevated by Recombinant IL-27 and Decreased by Anti-IL-27p28 Antibody

**Figure 3:**
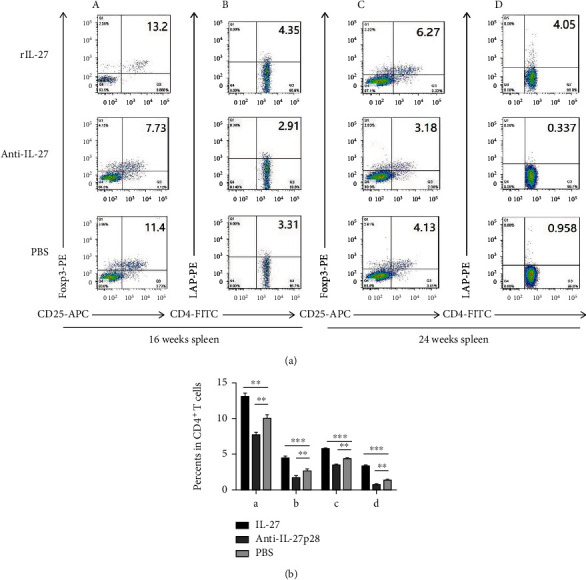
Recombinant IL-27 elevated the generation of Foxp3^+^ and LAP^+^ Tregs in the spleen of ApoE^−/−^ mice. (a) In the spleen, the fractions of Foxp3^+^ and LAP^+^ Tregs were measured by flow cytometry. (a) and (b) show the fractions of Foxp3^+^ Tregs (A) and LAP^+^ Tregs (B) during the initiation of atherosclerosis. (C) and (D) show the fractions of Foxp3^+^ Tregs (C) and LAP^+^ Tregs (D) during the progression of atherosclerosis. (b) Groups data are shown in histogram. ^∗∗∗^*P* < 0.001,^∗∗^*P* < 0.01, ^∗^*P* < 0.05.

**Figure 4:**
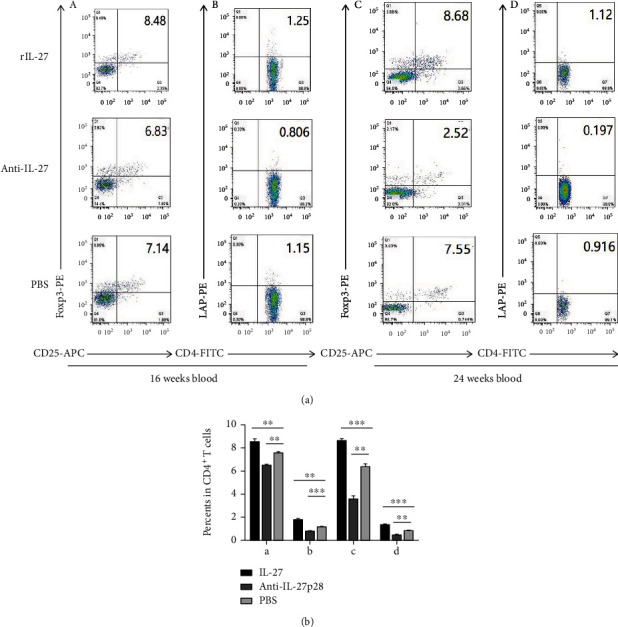
Recombinant IL-27 elevated the generation of Foxp3^+^ and LAP^+^ Tregs in the blood of ApoE^−/−^ mice. (a) In the blood, the fractions of Foxp3^+^ and LAP^+^ Tregs were measured by flow cytometry. (A) and (B) show the fractions of Foxp3^+^ Tregs (A) and LAP^+^ Tregs (B) during the initiation of atherosclerosis. (C) and (D) show the fractions of Foxp3^+^ Tregs (C) and LAP^+^ Tregs (D) during the progression of atherosclerosis. (b) Groups data are shown in histogram. ^∗∗∗^*P* < 0.001, ^∗∗^*P* < 0.01, ^∗^*P* < 0.05.

We determined the effect of recombinant IL-27 administration in ApoE mice. Using flow cytometry, we calculated the fractions of Foxp3^+^ Tregs and LAP^+^ Tregs in the spleen and peripheral blood of the ApoE^−/−^ mice. During the initiation of atherosclerosis, the generation of LAP^+^ Tregs (spleen: 4.4 ± 0.3% vs 2.6 ± 0.3%, respectively; *P* = 0.001; Figure 3) (blood: 1.7 ± 0.2% vs 1.13 ± 0.06%, respectively; *P* = 0.005; Figure 4) and Foxp3^+^ Tregs (spleen: 13.1 ± 0.5% vs 10.0 ± 0.5%, respectively; *P* = 0.002; Figure 3) (blood: 8.5 ± 0.3% vs 7.5 ± 0.1%, respectively; *P* = 0.006; Figure 4) was upregulated in the mice treated with IL-27 compared with those treated with PBS. In the mice treated with the antibody, LAP^+^ Treg (spleen: 1.7 ± 0.3% vs 2.6 ± 0.3%, respectively; *P* = 0.008; Figure 3) (blood: 0.75 ± 0.06% vs 1.13 ± 0.06%, respectively; *P* = 0.001; Figure 4), and Foxp3^+^ Treg (spleen: 7.68 ± 0.4% vs 10.0 ± 0.5%, respectively; *P* = 0.009; Figure 3) (blood: 6.4 ± 0.1% vs 7.5 ± 0.1%, respectively; *P* = 0.004; Figure 4) frequencies were lower than those in the mice treated with PBS. In groups modeling the progression of atherosclerosis, the proportions of LAP^+^ Treg (spleen: 3.3 ± 0.2% vs 1.34 ± 0.09%, respectively; *P* < 0.001; Figure 3) (blood: 1.34 ± 0.06% vs 0.81 ± 0.03%, respectively; *P* < 0.001; Figure 4), and Foxp3^+^ Treg (spleen: 5.7 ± 0.2% vs 4.3 ± 0.2%, respectively; *P* < 0.001; Figure 3) (blood: 8.6 ± 0.2% vs 6.3 ± 0.3%, respectively; *P* < 0.001; Figure 4) were upregulated in the mice treated with IL-27 compared with those treated with PBS. In the mice treated with the antibody, LAP^+^ Treg (spleen: 0.72 ± 0.10% vs 1.34 ± 0.09%, respectively; *P* = 0.004; Figure 3) (blood: 0.44 ± 0.07% vs 0.81 ± 0.03%, respectively; *P* = 0.005; Figure 4), and Foxp3^+^ Treg (spleen: 3.48 ± 0.09% vs 4.3 ± 0.2%, respectively; *P* = 0.009; Figure 3) (blood: 3.6 ± 0.3% vs 6.3 ± 0.3%, respectively; *P* = 0.006; Figure 4) numbers were lower than those in the mice treated with PBS. These data suggest that IL-27 may affect the initiation and progression of atherosclerosis by increasing the percentage of LAP^+^ and Foxp3^+^Treg.

### 4.5. In ApoE^−/−^ Mice, the Percentage of Th1 and Th17 Cells Was Reduced by Recombinant IL-27 and Increased by Anti-IL-27p28 Antibody

**Figure 5:**
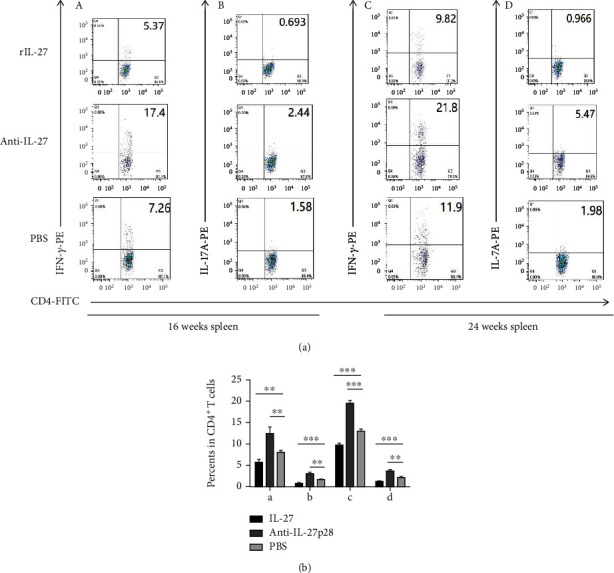
Recombinant IL-27 reduced the fractions of Th1 and Th17 cells in the spleen of ApoE^−/−^ mice. (a) In the spleen, the fractions of Th1 and Th17 cells were measured by flow cytometry. (A) and (B) show the fractions of Th1 (A) and Th17 cells (B) during the initiation of atherosclerosis. (C) and (D) show the fractions of Th1 (C) and Th17 cells (D) during the progression of atherosclerosis. (b) Groups data are shown in histogram. ^∗∗∗^*P* < 0.001, ^∗∗^*P* < 0.01, ^∗^*P* < 0.05.

**Figure 6:**
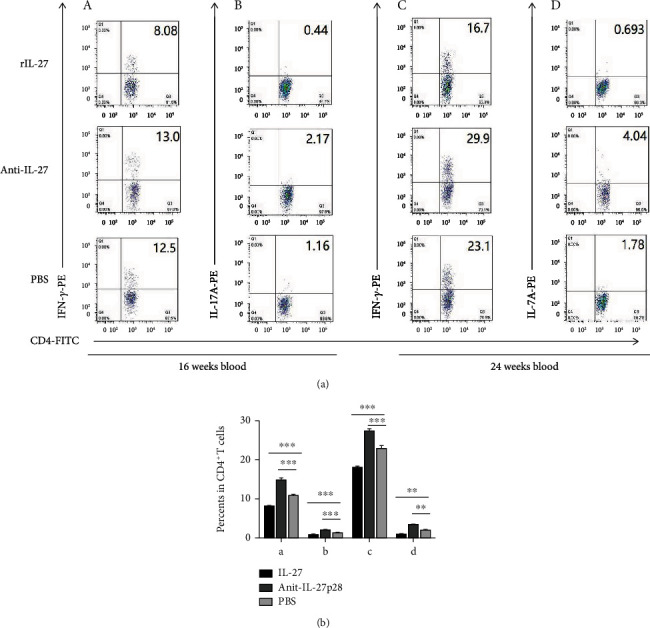
Recombinant IL-27 reduced the fractions of Th1 and Th17 cells in the blood of ApoE^−/−^ mice. (a) In the blood, the fractions of Th1 and Th17 cells were measured by flow cytometry. (A) and (B) show the fractions of Th1 (A) and Th17 cells (B) during the initiation of atherosclerosis. (C) and (D) show the fractions of Th1 (C) and Th17 cells (D) during the progression of atherosclerosis. (b) Groups data are shown in histogram. ^∗∗∗^*P* < 0.001, ^∗∗^*P* < 0.01, ^∗^*P* < 0.05.

To determine whether the changes in the number of Tregs affected effector T cells, Th1, Th2, and Th17 cells were also measured. In both the spleen and the peripheral blood, the mice treated with IL-27 during the initiation of atherosclerosis showed lower counts of Th1 (spleen: 5.7 ± 0.6% vs 8.0 ± 0.5%, respectively; *P* = 0.01; Figure 5) (blood: 7.9 ± 0.2% vs 10.7 ± 0.5%, respectively; *P* < 0.001; Figure 6), and Th17 cells (spleen: 0.74 ± 0.04% vs 1.63 ± 0.12%, respectively; *P* < 0.001; Figure 5) (blood: 0.72 ± 0.07% vs 1.27 ± 0.03%, respectively; *P* < 0.001; Figure 6) than the PBS-treated mice. In both the spleen and blood, higher counts of Th1 (spleen: 12.4 ± 1.5% vs 8.0 ± 0.5%, respectively; *P* = 0.007; Figure 5) (blood: 14.8 ± 0.6% vs 10.7 ± 0.5%, respectively; *P* < 0.001; Figure 6), and Th17 cells (spleen: 3.02 ± 0.28% vs 1.63 ± 0.12%, respectively; *P* = 0.009; Figure 5) (blood: 1.98 ± 0.13% vs 1.27 ± 0.03%, respectively; *P* < 0.001; Figure 6) were detected in the mice treated with the antibody than in the PBS-treated mice. In the groups modeling the progression of atherosclerosis, the mice treated with IL-27 showed lower counts of Th1 (spleen: 9.7 ± 0.5% vs 13.0 ± 0.5%, respectively; *P* < 0.001; Figure 5) (blood: 18.0 ± 0.4% vs 22.9 ± 0.7%, respectively; *P* = 0.001; Figure 6), and Th17 cells (spleen: 1.19 ± 0.06% vs 2.1 ± 0.1%, respectively; *P* < 0.001; Figure 5) (blood: 0.87 ± 0.06% vs 1.9 ± 0.2%, respectively; *P* = 0.005; Figure 6) than the PBS-treated mice. The mice treated with the antibody displayed higher counts of Th1 (spleen: 19.5 ± 0.7% vs 13.0 ± 0.5%, respectively; *P* < 0.001; Figure 5) (blood: 27.3 ± 0.7% vs 22.9 ± 0.7%, respectively; *P* < 0.001; Figure 6), and Th17 cells (spleen: 3.51 ± 0.47% vs 2.1 ± 0.1%, respectively; *P* = 0.01; Figure 5) (blood: 3.4 ± 0.3% vs 1.9 ± 0.2%, respectively; *P* = 0.005; Figure 6) than the PBS-treated mice. However, the percentage of Th2 cells were not significantly different among the groups (data not shown). These results suggest that IL-27 may affect the initiation and progression of atherosclerosis by decreasing the proportions of Th1 and Th17 effector T cells.

## 5. Recombinant IL-27 Reduced the Expression of IFN-*γ* and IL-17A While Increasing the Expression of TGF-*β*, IL-10, and Foxp3 in the Aorta

**Figure 7:**
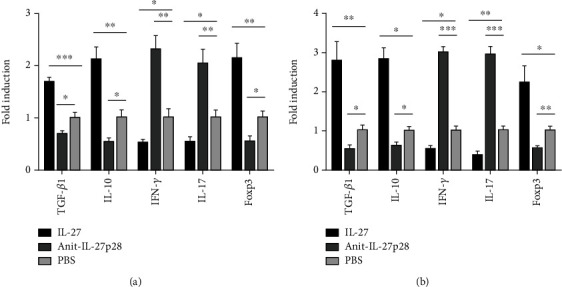
Recombinant IL-27 reduced the expression of IFN-*γ* and IL-17A while increasing the expression of TGF*-β_1_,* IL-10, and Foxp3 in the aorta. The expression of associated genes in the aorta during the initiation (a) and the progression (b) of atherosclerosis were detected by qPCR. Groups data are shown in histogram. ^∗∗∗^*P* < 0.001, ^∗∗^*P* < 0.01, ^∗^*P* < 0.05.

To determine the changes in mRNA levels, the expression of associated genes in the aorta during the initiation of atherosclerosis was detected by qPCR (Figure 7(a)). TGF-*β* (1.68 ± 0.10 − fold vs 1.00 ± 0.09 − fold, respectively; *P* < 0.001), Foxp3 (2.1 ± 0.3 − fold vs 1.00 ± 0.10 − fold, respectively; *P* = 0.005), and IL-10 (2.1 ± 0.2 − fold vs 1.00 ± 0.07 − fold, respectively; *P* = 0.003) levels were higher in the mice treated with IL-27 than in those treated with PBS. IFN-*γ* (0.52 ± 0.06 − fold vs 1.00 ± 0.08 − fold, respectively; *P* = 0.008), and IL-17 (0.60 ± 0.10 − fold vs 1.00 ± 0.03 − fold, respectively; *P* = 0.005) were suppressed in the mice treated with IL-27 compared with those treated with PBS. When anti-IL-27p28-Ab was administered, TGF-*β* (0.68 ± 0.07 − fold vs 1.00 ± 0.09 − fold, respectively; *P* = 0.007), Foxp3 (0.5 ± 0.1 − fold vs 1.00 ± 0.10 − fold, respectively; *P* = 0.004), and IL-10 (0.54 ± 0.08 − fold vs 1.00 ± 0.07 − fold, respectively; *P* = 0.005) levels were detected to be lower than the levels after PBS administration. IFN-*γ* (2.3 ± 0.3 − fold vs 1.00 ± 0.08 − fold, respectively; *P* = 0.003), and IL-17 (2.00 ± 0.30 vs 1.00 ± 0.03 − fold, *P* = 0.004) levels were increased in the mice treated with anti-IL-27p28-Ab compared with those treated with PBS. In the groups modeling the progression of atherosclerosis (Figure 7(b)), TGF-*β* (2.80 ± 0.50 − fold vs 1.00 ± 0.08 − fold, respectively; *P* = 0.005), Foxp3 (2.23 ± 0.43 − fold vs 1.00 ± 0.06 − fold, respectively; *P* = 0.007), and IL-10 (2.56 ± 0.57 − fold vs 1.00 ± 0.10 − fold, respectively; *P* = 0.007) showed higher levels in the mice treated with IL-27 than in those treated with PBS. IFN-*γ* (0.54 ± 0.08 − fold vs 1.00 ± 0.06 − fold, respectively; *P* < 0.001), and IL-17 (0.38 ± 0.10 − fold vs 1.00 ± 0.08 − fold, respectively; *P* < 0.001) expression was inhibited in the mice treated with IL-27 compared with those treated with PBS. TGF-*β* (0.53 ± 0.11 − fold vs 1.00 ± 0.08 − fold, respectively; *P* = 0.004), Foxp3 (0.55 ± 0.06 − fold vs 1.00 ± 0.06 − fold, respectively; P = 0.005), and IL-10 (0.61 ± 0.10 − fold vs 1.00 ± 0.10 − fold, respectively; *P* = 0.009) levels were lower in the mice treated with anti-IL-27p28-Ab than in those treated with PBS. IFN-*γ* (3.00 ± 0.16 − fold vs 1.00 ± 0.06 − fold, respectively; *P* < 0.001), and IL-17 (2.94 ± 0.21 − fold vs 1.00 ± 0.08 − fold, respectively; *P* < 0.001) levels were increased in the mice treated with anti-IL-27p28-Ab compared with those treated with PBS. In sum, these data showed that recombinant IL-27 increased Treg-associated cytokine expression and decreased Th1 and Th17 associated cytokine expression in the aorta.

### 5.1. Recombinant IL-27 Reduced the Levels of IFN-*γ* and IL-17A in the Peripheral Blood Plasma While Increasing the Levels of TGF-*β* and IL-10

**Figure 8:**
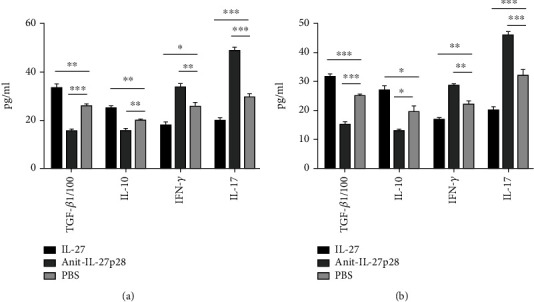
Recombinant IL-27 reduced the levels of IFN-*γ* and IL-17A in the peripheral blood plasma *while increasing the levels of* TGF-*β* and IL-10. Related cytokines level in mice plasma was detected by ELISA. Data from both the initiation (a) and the progression (b) of atherosclerosis were shown in histogram. The meaning of “TGF-b1/100” was *the level of* TGF-*β* divided by 100. *Because the levels of* TGF-*β* were much higher than *the levels of* IL-10, IFN-*γ*, *and IL-17A, we used* “TGF-b1/100” in this Figure. ^∗∗∗^*P* < 0.001, ^∗∗^*P* < 0.01, ^∗^*P* < 0.05.

Related cytokines were detected by ELISA (Figure 8). During the initiation of atherosclerosis, (Figure 8(a)) TGF-*β*_1_ (3,309 ± 196 vs 2,567 ± 115 pg/mL, respectively; *P* = 0.008) and IL-10 (24.95 ± 1.07 vs 19.70 ± 0.63 pg/mL, respectively; *P* = 0.002) expressions were promoted in the mice treated with IL-27 compared with those treated with PBS, while IFN-*γ* (17.85 ± 1.47 vs 25.50 ± 2.02 pg/mL, respectively; *P* = 0.01) and IL-17 (19.69 ± 1.45 vs 29.43 ± 1.50 pg/mL, respectively; *P* = 0.001) levels were reduced. TGF-*β*1 (1,526 ± 115 vs 2,567 ± 115 pg/mL, respectively; *P* = 0.005) and IL-10 (15.65 ± 1.08 vs 19.70 ± 0.63 pg/mL, respectively; *P* = 0.006) levels were decreased while IFN-*γ* (33.63 ± 1.57 vs 25.50 ± 2.02 pg/mL, respectively; *P* = 0.004) and IL-17 (48.52 ± 1.63 vs 29.43 ± 1.50 pg/mL, respectively; *P* = 0.002) levels were increased in the mice treated with the anti-IL-27 antibody compared with those treated with PBS. During the progression of atherosclerosis, (Figure 8(b)) TGF-*β* (3,159 ± 106 vs 2,483 ± 68 pg/mL, respectively; *P* < 0.001) and IL-10 (26.78 ± 1.74 vs 19.37 ± 2.19 pg/mL, respectively; *P* = 0.02) were *showed higher levels* in the mice treated with IL-*27 than in those treated with PBS, while* IFN-*γ* (16.67 ± 0.90 vs 21.88 ± 1.36 pg/mL, respectively; *P* = 0.01) and IL-17 (19.98 ± 1.28 vs 31.85 ± 2.24 pg/mL, respectively; *P* = 0.001) were displayed lower levels. Compared with those in the PBS group, the TGF-*β* (1,493 ± 110 vs 2,483 ± 68 pg/mL, respectively; *P* = 0.005) and IL-10 (12.81 ± 0.57 vs 19.37 ± 2.19 pg/mL, respectively; *P* = 0.008) levels in the antibody group were decreased, while *the* IFN-*γ* (28.42 ± 0.76 vs 21.88 ± 1.36 pg/mL, respectively; *P* = 0.004) and IL-17 (19.98 ± 1.28 vs 19.98 ± 1.28 pg/mL, respectively; *P* = 0.004) levels were increased. Collectively, these data showed that recombinant IL-27 increased Treg-associated cytokine levels and decreased Th1 and Th17 associated cytokine levels.

### 5.2. IL-27-tDCs Showed the Tolerogenic Phenotype

**Figure 9:**
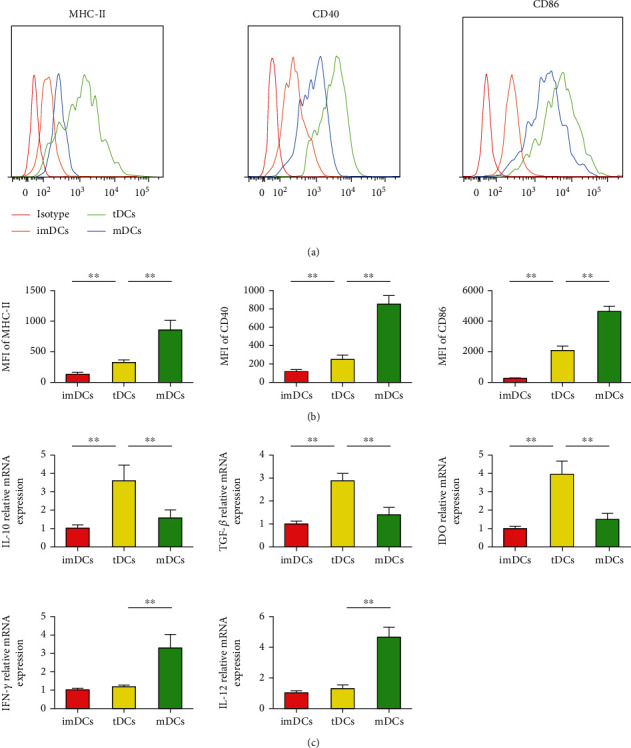
Tolerogenic dendritic cells (tDCs) increase the percentage of regulatory T cells (Tregs) in vitro. (a), Splenic CD4^+^ T cells (1 × 10^6^ cells/mL) were cultured for 3 days in the presence of medium alone or combined with immature DCs (imDCs), mature DCs (mDCs), or IL-27-tDCs (2 × 10^5^ cells/mL). The cells were labeled 72 hours later with anti-CD4, anti-CD25, and anti-Foxp3 and analyzed by FACS. FACS data are representative of 1 of 6 to 8 independent experiments (each coculture preparation was prepared from a different mouse). (b) Graphs represent the average percentages of Tregs in CD4^+^ splenic T cells of different groups. (c) Analysis of the mRNA levels of interferon-*γ* (IFN-*γ*), IL-17A, Foxp3, interleukin (IL)–10, and transforming growth factor-*β* (TGF-*β*) in different groups. No DCs group *n* = 6, imDCs group *n* = 8, mDCs group *n* = 8, and tDCs group *n* = 8. ^∗∗^*P* < 0.01.

DC maturation is characterized by expansion of antigen-presenting molecules (such as MHC II, CD40, and CD86). DCs were stained with isotype control antibody, anti-MHC-II antibody, anti-CD40, and anti-CD86 antibodies, followed by FACS analysis (*P* < 0.05, Figure 9(a)), and then the mean fluorescence intensity (MFI) was detected. Compared with the mDC group, the IL-27-tDC group displayed lower MFI of MHC-II, CD40, and CD86 (*P* < 0.05, Figure 9(b)). As shown in Figure 9(c), DCs loaded with IL-27 and LPS produced lower mRNA levels of IFN-*γ* and IL-12, as well as higher mRNA levels of IL-10, TGF-*β*, and IDO, than the mDCs. These results together demonstrate that IL-27 plus LPS–treated DCs display a tolerogenic phenotype.

### 5.3. IL-27-tDCs Showed the Ability to Induce Tolerance when Cocultured with CD4^+^ T Cells

**Figure 10:**
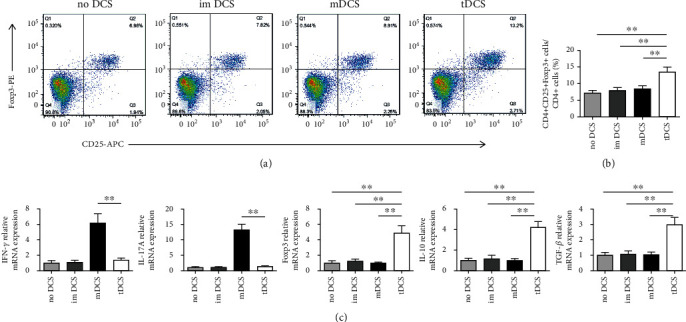
Interleukin (IL)–27 plus lipopolysaccharide (LPS)–treated dendritic cells (DCs) display tolerogenic properties. (a) Bone marrow–derived DCs (2 × 10^5^ cells/well) were cultured in the absence of stimulus (immature DCs [imDCs]) or in the presence of 100 ng/mL LPS (mature DCs [mDCs]) or 100 ng/mL LPS and 30 ng/mL IL-27 (tolerogenic DCs [tDCs]). DCs were stained with isotype control antibodies or with specific antibodies against major histocompatibility complex class II (MHC-II), CD40, and CD86 and analyzed by fluorescence-activated cell sorting (FACS). (b) Mean fluorescence intensities (MFIs) for MHC-II, CD40, and CD86 were investigated. (c) Analysis of the mRNA levels in different DCs groups. *n* = 6 per group. ^∗∗^*P* < 0.01.

To investigate the effect of IL-27-tDCs on Treg cells, CD4^+^ T cells were cocultured with different types of DCs in vitro, respectively. Compared with no DCs, imDCs and mDCs, IL-27-tDCs remarkedly increased the percentage of CD4^+^CD25^+^Foxp3^+^ Tregs in the CD4^+^ cells (*P* < 0.05, Figures 10(a) and 10(b)), and the mRNA level of Foxp3 in the IL-27-tDC group was significantly higher than that in the other three groups (*P* < 0.05, Figure 10(c)). Furthermore, IL-27-tDCs significantly inhibited IFN-*γ* and IL-17A mRNA levels compared with the mDCs. In contrast, mRNA levels of IL-10 and TGF-*β* were significantly enhanced in the IL-27-tDC group (*P* < 0.05, Figure 10(c)). In summary, the above results showed that IL-27-tDCs exhibited the ability to induce tolerance when cocultured with CD4^+^ T cells.

### 5.4. IL-27-tDCs Alleviate Atherosclerosis in ApoE^−/−^ Mice

**Figure 11:**
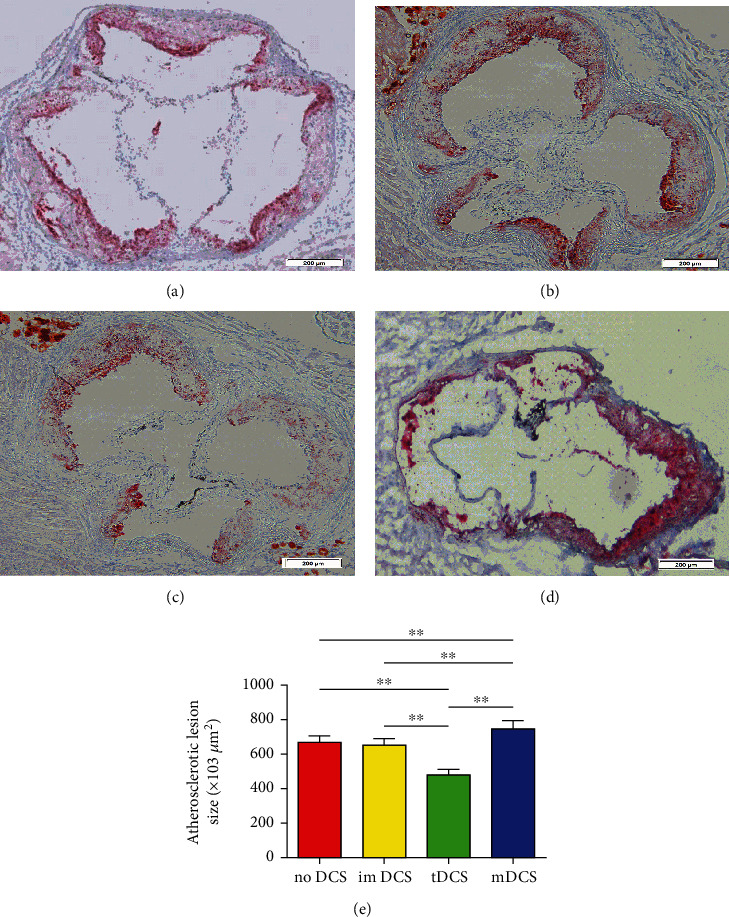
IL-27-tDCs ameliorate atherosclerosis in ApoE^−/−^ mice. (a) The atherosclerotic lesion size in the no DC group. (b) The atherosclerotic lesion size in the imDC group. (c) The atherosclerotic lesion size in the IL-27-tDC group. (d) The atherosclerotic lesion size in the mDC group. (e) The atherosclerotic lesion size was counted by Image Pro. *n* = 6 per group. The black bar represents 100 *μ*m. ^∗∗^*P* < 0.001.

To test the role of IL-27-tDCs on atherosclerosis, 6-week-old mice were given a high-fat diet for 10 weeks to induce plaque formation in aortic root, and then no DCs (Figure 11(a)), imDCs (Figure 11(b)), IL-27-tDCs (Figure 11(c)), and mDCs (Figure 11(d)) were adoptive transferred to atherosclerotic mice. Sections from the aortic root were stained with oil red O and hematoxylin to observe the plaques. As expected, adoptive transfer of IL-27-tDCs significantly reduced atherosclerotic plaque size compared with mDCs (Figure 11(e)). However, the plaque size of the mDC group was even significantly increased compared with the imDC and the IL-27-tDC group (Figure 11(e)). These results indicate that adoptive transfer of IL-27-tDCs can significantly improve atherosclerosis, while adoptive transfer of mDCs can remarkedly aggravate atherosclerosis.

## 6. Discussion

Atherosclerosis, a chronic inflammatory disease of the arterial wall, has been reported to be associated with various inflammatory cytokines, such as IL-10 and TGF-*β*, which are associated with Tregs [25] as well as IFN-*γ,* TNF*-α,* and IL-17, which are associated with Th1 and Th17 cells [26]. Compelling experimental data suggest that shifting the Treg/effector T cell balance toward Tregs may be a possible therapeutic approach for atherosclerotic disease. For instance, we previously reported that intranasal immunization with heat shock protein 60-induced Tregs inhibited early atherosclerosis [14]. We also reported that Tregs induced by nasal-oxidized low-density lipoprotein suppressed the effector T cell response and attenuated atherosclerosis [15]. However, the role of Tregs in human atherosclerotic disease has not been fully elucidated.

Several immunological therapies, such as the use of an FcR-nonbinding anti-CD3 monoclonal antibody [27, 28], an IL-2/anti-IL-2 monoclonal antibody complex [29], their combination [30], and the active form of vitamin D_3_, have been shown to be valid approaches to prevent atherosclerosis by increasing Treg numbers [31]. However, traditional immunological approaches to attenuate the immunoinflammatory component of atherosclerosis are limited, particularly in established atherosclerosis. For example, anti-CD3 treatment in mice induces CD4^+^LAP^+^ Tregs and attenuates the initiation of atherosclerosis but not the progression of established atherosclerosis [29]. According to the study by Dinh et al., we modified published short-term treatment protocols to make the treatment suitable for inducing long-term elevations in Treg numbers [29]. A previous report of Koltsova et al. showed that ldlr^−/−^ mice transplanted with IL27ra^−/−^ bone marrow develop significantly larger atherosclerotic lesions in the aortic roots, aortic arches, and abdominal aortas than those transplanted with wild-type bone marrow [32]. These findings suggest that IL-27 may be a promising therapeutic approach for attenuating atherosclerosis. Indeed, our results showed that the administration of recombinant IL-27 inhibited both the initiation and the progression of atherosclerotic plaques in ApoE^−/−^ mice. Mice received recombinant IL-27 treatment showed a smaller aortic plaque burden, and we are the first to report that mice received an anti-IL-27p28 antibody grew larger plaques. Based on these findings, targeting IL-27-IL27 receptor axis could be a potential strategy for the prevention or therapy of atherosclerosis.

IL-27 functions as a pleiotropic cytokine under both physiological and pathological conditions. IL-27 production is triggered by various TLR agonists, including lipopolysaccharide (LPS), poly (I:C), CPG, and Gram-negative and Gram-positive bacteria. IFN-*γ* regulatory factors (IRFs), including IRF1, IRF3, IRF7, and IRF8, enhanced IL-27 production [33–37]. IL-27 was first recognized as a proinflammatory cytokine [38–40]. IL-27 were observed to be important in Th1 responses against those parasites. Moreover, studies have found that IL-27 inhibited the expression of Foxp3, CD25, and CTLA4 through inhibiting STAT3 and therefore suppressed the generation of Tregs [41, 42]. In IL-27 transgenic mice, both CD4^+^CD25^+^ T cell models and bone marrow transfer models showed deficiencies in Treg differentiation [41].

However, recent studies have revealed the complexity of IL-27 function. Subsequent studies have shown that IL-27 is a negative regulator of IL-2 in vitro and can restrict the development of immune responses [43–45]. Additionally, in in vivo parasite infection models, IL-27 inhibits IFN-*γ* production and attenuates disease pathology associated with Toxoplasma gondii, Trypanosoma cruzi, Plasmodium berghei, Leishmania donovani, and malaria [46–49]. The same trend was observed for Tregs. During T. gondii infection, IL-27 promoted T-bet and CXCR3 expression in Tregs through STAT1, STAT3, and STAT5 [44]. In our study, the anti-inflammatory properties of IL-27 resulted in increased generation of Foxp3^+^ and LAP^+^ Tregs and reduced the number of Th1 and Th17 cells in ApoE^−/−^ mice. This outcome is consistent with the findings of a study by Koltsova et al., who reported that the absence of anti-inflammatory IL-27 signaling results in lower Treg and higher Th1 and Th17 cell counts in the model of ldlr^−/−^ mice transplanted with Il27ra^−/−^ bone marrow [32]. Furthermore, it was reported that in the absence of the IL-27 receptor, macrophage activation in vitro increased modified low-density lipoprotein uptake and proinflammatory cytokine production [50]. Of note, our study is the first to evaluate the effect of the administration of an anti-IL-27p28 antibody in ApoE^−/−^ mice and the role of Recombinant IL-27 in LAP^+^ Tregs.

Importantly, targeting classic Tregs for treatment is limited by the detection of Foxp3 on activated cells [51]. Except for Foxp3, few reliable and specific markers have been reported for distinguishing Treg subpopulations. Foxp3 is crucial for the development and maintenance of the suppressive Treg lineage. However, it was reported that Helios should be a better marker of activated Tregs expressing Glycoprotein A repetitions predominant (GARP/LRRC32)/LAP than Foxp3 [52]. LAP and TGF-*β* form inactive complexes known as latent TGF-*β* complexes on the surface of T cells. These complexes can be cleaved to release active TGF-*β* [53]. GARP plays a critical role in the formation and expression of latent TGF-*β* complexes at the cell surface by anchoring the complexes to the cell membrane [52]. These new markers all have the characteristic of being located on the cell membrane, making them more valuable than Foxp3 in translational medicine. However, more experiments in different situations and diseases are needed.

Our recent studies showed that CD4^+^LAP^+^ and CD4^+^CD25^+^ Tregs are suppressed in ACS [13] and that CD4^+^LAP^+^ and CD4^+^CD25^+^Foxp3^+^ T cells induced by nasal-oxidized low-density lipoprotein suppress effector T cell responses and attenuate atherosclerosis in ApoE^−/−^ mice [15]. Moreover, Gabriely et al. reported that an anti-LAP antibody represents a potential approach for cancer immunotherapy by decreasing LAP^+^ Treg numbers [54]. All these studies have shown that LAP acts as a reliable marker of Tregs in many situations. Therefore, CD4^+^LAP^+^ Tregs were of interest to us. CD4^+^LAP^+^ Tregs were previously identified as a new subset of Tregs [12]. LAP^+^ population is relatively small, however, several studies have mentioned that CD4^+^LAP^+^ Tregs share many properties with Foxp3^+^ Tregs [15, 16, 55]. Previous data suggested that TGF-*β* plays a crucial role in the suppressive function of natural Tregs (nTregs) and CD4^+^LAP^+^ Tregs [12, 56, 57]. We are the first to address whether IL-27-induced CD4^+^LAP^+^ Tregs and CD4^+^LAP^+^ Tregs play key roles in atherogenesis. However, accurate mechanisms describing how IL-27 affects T cells in vivo remain elusive.

It is known that naive CD4^+^ T cells differentiate into effector T cells such as Th1, Th2, and Th17 cells, all of which critically affect atherogenesis in both humans and mice after antigen presentation or activation by macrophages or DCs [26]. Th1 cells producing high amounts of IFN-*γ* and Th17 cells producing high amounts of IL-17 are the most common pathogenic T cells in atherosclerosis. IFN-*γ* is reported to initiate the activation of monocytes/macrophages and DCs, resulting in pathogenic Th1 response continuation [58]. The proatherogenic role of Th17 cells has also been demonstrated [59]. Our data showed that the administration of recombinant IL-27 led to smaller percentage of Th1 and Th17 cells. The decline in these proatherogenic T cell populations should be responsible for the attenuated atherosclerosis. In addition, our data indicate that the administration of recombinant IL-27 suppresses IFN-*γ* and IL-17 secreted by effector T cells. Our results confirm that Th1 and Th17 cells play proatherogenic roles through the production of IFN-*γ* and IL-17, respectively. Furthermore, our data showed that body weight and lipid profiles were not significantly different between IL-27-treated mice and PBS-treated mice. These results suggested that IL-27 suppressed the development of atherosclerosis independent of body mass index and lipid levels in mice.

As each coin has two sides, DCs are professional antigen–presenting cells that regulate both immunity and tolerance [17, 18]. Mature DCs are immunogenic, while semimature DCs are tolerogenic [19]. After MI, due to secondary myocardial injury, autoimmune response to cardiac myosin or Troponin I is concerned with adverse clinical outcomes [60, 61]. A previous report showed that myosin-loaded tDCs attenuate autoimmune myocarditis [22]. In line with this study, our research detected that thymic stromal lymphopoietin–conditioned DCs ameliorate atherosclerosis development via inducing differentiation of Tregs [16]. In sum, the above publications demonstrated that tDCs play a protective part in these models. Of note, in hypercholesterolemic mice, atherosclerosis was accelerated by administration of LDL-induced DCs and attenuated by immunotherapy with DCs treated with ApoB100 and IL-10, indicating that atherosclerosis could be modulated by modifying the maturation of DCs [23, 62]. In the present study, the MFI of CD40, MHC-II, and CD86 were markedly decreased in the IL-27-tDC group compared with mDC group, suggesting that IL-27 impeded the maturation process of DCs. In vitro, we also demonstrated that IL-27-tDCs exhibited the ability to induce tolerance. Furthermore, adoptive transmission of IL-27-tDCs to ApoE^−/−^mice displayed that atherosclerotic plaques were reduced. This result indicates that IL-27 can improve atherosclerosis by induction of tDCs. However, the exact mechanism of how IL-27 induce tolerance of DCs are still need to be elucidated.

## 7. Conclusion

In summary, our study is the first to highlight that LAP^+^ Tregs play a crucial role in the IL-27 signaling that limits atherosclerosis. We reported that the administration of IL-27 prevents both the initiation and the progression of atherosclerosis by inducing LAP^+^ and Foxp3^+^ Tregs. We also demonstrated that DCs treated with IL-27 display a tolerogenic phenotype and that IL-27–treated tDCs are likely to play a protective role during in atherosclerosis, suggesting that IL-27 or adoptive transfer of IL-27 loaded tDCs may provide a novel approach for the treatment of atherosclerosis in the future.

## Figures and Tables

**Table 1:** 

Molecule	Sequence (5′-3′)
TGF-*β*_1_	GTGTGGAGCAACATGTGGAACTCTA
	TTGGTTCAGCCACTGCCGTA

IL-10	ATGCTGCCTGCTCTTACTGACTG
	CCCAAGTAACCCTTAAAGTCCTGC

IFN-*γ*	AGAGCCAGATTATCTCTTTCTACCTCAG
	CCTTTTTCGCCTTGCTGTTG

IL-17	GAAGCTCAGTGCCGCCA
	TTCATGTGGTGGTCCAGCTTT

Foxp3	CTCATGATAGTGCCTGTGTCCTCAA
	AGGGCCAGCATAGGTGCAAG

IDO	CAGCTTCTCCTGCAATCAAAGCA
	TGCGAGGTGGAACTTTCTCACAGA

IL-12	ATCGTTTTGCTGGTGTCTCC
	CTTTGTGGCAGGTGTACTGG

GAPDH	GTGCTGAGTATGTCGTGGAG
	GTCTTCTGAGTGGCAGTGAT

## Data Availability

The data used to support the findings of this study are available from the corresponding author upon request.

## Abbreviations

IL: Interleukin
ACS: Acute coronary syndrome
Treg: Regulatory T cell
LAP: Latency-associated peptide
Th: T helper
TNF: Tumor necrosis factor
TGF: Transforming growth factor
IFN: Interferon
EBI3: Epstein-Barr virus-induced gene3
NK: Natural killer
Tfh: T follicular helper
SAP: Stable angina pectoris
PMBC: Peripheral blood mononuclear cell
PBS: Phosphate-buffered saline
OCT: Optimum cutting temperature
DC: Dendritic cell
tDCs: Tolerogenic dendritic cells
mDCs: Mature dendritic cells
GARP: Glycoprotein a repetitions predominant.

## Supplementary Materials

Supplementary Materials Figure S1: after fed a high-fat diet for 8 weeks, aortic sinus atherosclerotic lesion size (A) in the 166, 500, 1500 ng/mL IL-27 and PBS groups. The fractions of Foxp3^+^ Tregs (B), LAP^+^ Tregs (C), Th1 (D), and Th17 (E) cells in the peripheral blood of the ApoE^−/−^ mice were calculated in the 166, 500, 1500 ng/mL IL-27 and PBS groups. Aortic sinus atherosclerotic lesion size (F) in the 16.6, 50, and 150 *μ*g/mL anti-IL-27p28 antibody and PBS groups. The fractions of Foxp3^+^ Tregs (G), LAP^+^ Tregs (H), Th1 (I), and Th17 (J) cells in the peripheral blood of the ApoE^−/−^ mice were calculated in the 16.6, 50, and 150 *μ*g/mL anti-IL-27p28 antibody and PBS groups. *n* = 6 per group. ^∗^*P* < 0.05 and ^∗∗^*P* < 0.01.
