# Supplementary material for: Interleukin-27 Ameliorates Atherosclerosis in ApoE−/− Mice through Regulatory T Cell Augmentation and Dendritic Cell Tolerance
Source: Mediators Inflamm. 2022 Nov 11;2022:2054879. doi: 10.1155/2022/2054879 (PMC9674420; doi:10.1155/2022/2054879)
Supplement: Supplementary Materials — Figure S1: after fed a high-fat diet for 8 weeks, aortic sinus atherosclerotic lesion size (A) in the 166, 500, 1500 ng/mL IL-27 and PBS groups. The fractions of Foxp3+ Tregs (B), LAP+ Tregs (C), Th1 (D), and Th17 (E) cells in the peripheral blood of the ApoE−/− mice were calculated in the 166, 500, 1500 ng/mL IL-27 and PBS groups. Aortic sinus atherosclerotic lesion size (F) in the 16.6, 50, and 150 μg/mL anti-IL-27p28 antibody and PBS groups. The fractions of Foxp3+ Tregs (G), LAP+ Tregs (H), Th1 (I), and Th17 (J) cells in the peripheral blood of the ApoE−/− mice were calculated in the 16.6, 50, and 150 μg/mL anti-IL-27p28 antibody and PBS groups. n = 6 per group. ∗P < 0.05 and ∗∗P < 0.01. [file 2054879.f1.zip › Supplementary figure legend (1) (2).docx]

**Figure S1** After fed a high-fat diet for 8 weeks, aortic sinus atherosclerotic lesion size (A) in the 166, 500, 1500 ng/mL IL-27 and PBS groups. The fractions of Foxp3^+^ Tregs (B), LAP^+^ Tregs (C), Th1 (D), and Th17 (E) cells in the peripheral blood of the ApoE^-/-^ mice were calculated in the 166, 500, 1500 ng/mL IL-27 and PBS groups. Aortic sinus atherosclerotic lesion size (F) in the 16.6, 50, and 150 µg/mL anti-IL-27p28 antibody and PBS groups. The fractions of Foxp3^+^ Tregs (G), LAP^+^ Tregs (H), Th1 (I), and Th17 (J) cells in the peripheral blood of the ApoE^-/-^ mice were calculated in the 16.6, 50, and 150 µg/mL anti-IL-27p28 antibody and PBS groups. n=6 per group. *P<0.05 and **P<0.01.
