# Supplementary material for: Interleukin-27 Ameliorates Atherosclerosis in ApoE−/− Mice through Regulatory T Cell Augmentation and Dendritic Cell Tolerance
Source: Mediators Inflamm. 2022 Nov 11;2022:2054879. doi: 10.1155/2022/2054879 (PMC9674420; doi:10.1155/2022/2054879)
Supplement: Supplementary Materials — Figure S1: after fed a high-fat diet for 8 weeks, aortic sinus atherosclerotic lesion size (A) in the 166, 500, 1500 ng/mL IL-27 and PBS groups. The fractions of Foxp3+ Tregs (B), LAP+ Tregs (C), Th1 (D), and Th17 (E) cells in the peripheral blood of the ApoE−/− mice were calculated in the 166, 500, 1500 ng/mL IL-27 and PBS groups. Aortic sinus atherosclerotic lesion size (F) in the 16.6, 50, and 150 μg/mL anti-IL-27p28 antibody and PBS groups. The fractions of Foxp3+ Tregs (G), LAP+ Tregs (H), Th1 (I), and Th17 (J) cells in the peripheral blood of the ApoE−/− mice were calculated in the 16.6, 50, and 150 μg/mL anti-IL-27p28 antibody and PBS groups. n = 6 per group. ∗P < 0.05 and ∗∗P < 0.01. [file 2054879.f1.zip › supplementary figure.docx]

Please note that the supplementary file is not uploaded in the "supplementary files section" in the formats ppt, XML.

Hence, the supplementary file of the manuscript is uploaded in the figure & table.
